# Charged hadron fragmentation functions from collider data: NNPDF Collaboration

**DOI:** 10.1140/epjc/s10052-018-6130-4

**Published:** 2018-08-16

**Authors:** V. Bertone, N. P. Hartland, E. R. Nocera, J. Rojo, L. Rottoli

**Affiliations:** 1 Department of Physics and Astronomy, Vrije Universiteit Amsterdam, 1081 HV Amsterdam, The Netherlands; 2 Nikhef Theory Group, Science Park 105, 1098 XG Amsterdam, The Netherlands; 3 Higgs Centre for Theoretical Physics, School of Physics and Astronomy, University of Edinburgh, Edinburgh, EH9 3FD UK; 4 Clarendon Laboratory, Rudolf Peierls Centre for Theoretical Physics, University of Oxford, Parks Road, Oxford, OX1 3PU UK

## Abstract

We present NNFF1.1h, a new determination of unidentified charged-hadron fragmentation functions (FFs) and their uncertainties. Experimental measurements of transverse-momentum distributions for charged-hadron production in proton-(anti)proton collisions at the Tevatron and at the LHC are used to constrain a set of FFs originally determined from electron–positron annihilation data. Our analysis is performed at next-to-leading order in perturbative quantum chromodynamics. We find that the hadron-collider data is consistent with the electron–positron data and that it significantly constrains the gluon FF. We verify the reliability of our results upon our choice of the kinematic cut in the hadron transverse momentum applied to the hadron-collider data and their consistency with NNFF1.0, our previous determination of the FFs of charged pions, kaons, and protons/antiprotons.

## Introduction

The determination of the collinear unpolarised fragmentation functions (FFs) of neutral and charged hadrons has been a topic of active research in the last decade [1]. FFs describe how coloured partons are turned into hadrons and can be regarded as the final-state counterparts of the parton distribution functions (PDFs) [2]. Since FFs are non-perturbative quantities in quantum chromodynamics (QCD), they need to be determined from an analysis of experimental data.

The recent interest in FFs stems from the copious amount of precise measurements that have been and are currently being collected for different processes at various centre-of-mass energies. These include data for hadron production in: single-inclusive $e^+e^-$ annihilation (SIA) (recently measured by BELLE [3, 4] and BABAR [5]), semi-inclusive deep-inelastic scattering (SIDIS) (recently measured by HERMES [6] and COMPASS [7, 8]) and proton-(anti)proton (*pp*) collisions (measured, e.g., by CDF [9, 10] at the Tevatron, STAR [11] and PHENIX [12] at RHIC and CMS [13, 14] and ALICE [15] at the LHC). These measurements span a wide range in energy and momentum fraction and are sensitive to different partonic combinations. Therefore, they offer a unique opportunity to determine FFs with an unprecedented accuracy.

Several analyses exploited some of these measurements to constrain the FFs of the lightest charged hadrons, *i.e.* $\pi ^\pm$, $K^\pm$, and $p/\overline{p}$. Among the most recent studies, the HKKS16 [16], JAM16 [17], and NNFF1.0 [18] analyses are based on SIA data only. A global determination of the charged pion and kaon FFs was carried out in Refs. [19, 20], where SIDIS and *pp* data was also included. The FFs of heavier hadrons, such as $D^*$ [21, 22], $\Lambda$ [23, 24] and $\eta$ [25], were also studied, mostly from SIA measurements, although available data is in general scarcer than for light hadrons.

A further family of FFs with phenomenological relevance are those of the unidentified charged hadrons. They can be regarded as the sum of the FFs of all charged hadrons that can be produced in the fragmentation of a given parton. These FFs find application, for example, in the description of the charged-particle spectra measured in proton-ion and ion-ion collisions, which are actively investigated by current RHIC [26] and LHC [27] heavy-ion programs.

Despite the fair amount of measurements sensitive to unidentified charged-hadron FFs, they have received less attention as compared to identified charged-hadron FFs. As a matter of fact, only a few extractions have been carried out until recently [28–31]. The analysis of Ref. [31] is the only fit based on SIA, SIDIS and *pp* data, while all others are based on SIA data only. These FF sets were extracted some time ago from older measurements and it has been shown [32] that they do not describe the more recent transverse-momentum charged-particle spectra measured at the Tevatron and the LHC.

New analyses of unidentified charged-hadron FFs have been presented recently [33, 34] based only upon SIA data. In particular, the determination in Ref. [33] was performed using the NNPDF fitting methodology [35–37] designed to provide a statistically sound representation of experimental uncertainties with minimal procedural bias. As the SIA dataset used in this analysis has little power to constrain the gluon FF, the resulting gluon distribution was found to be affected by large uncertainties, within which the discrepancy in the description of *pp* data reported in Ref. [32] could be mitigated.

The purpose of this paper is to complement the analysis of Ref. [33] with the most recent measurements of the transverse-momentum charged-hadron spectra in *pp* collisions. These measurements are directly sensitive to the so far poorly known gluon fragmentation, therefore their inclusion in a fit is expected to provide a stringent constraint on this distribution. The *pp* data is included by means of Bayesian reweighting [38, 39]. The result, NNFF1.1h, is a new set of FFs for unidentified charged hadrons from a global analysis of SIA and *pp* data.

The paper is organised as follows. In Sect. 2, we present the data set included in this analysis and discuss how the theoretical predictions of the corresponding observables are computed. In Sect. 3, we present the main results of our analysis. Specifically, in Sect. 3.1 we discuss the quality of the fit and the impact of the hadron-collider data on the FFs; in Sect. 3.2 we motivate our choice for the kinematic cut on the transverse-momentum of the final-state hadron applied to *pp* data; and in Sect. 3.3 we assess the consistency of the current determination with NNFF1.0 [18], our previous analysis of FFs for charged pions, kaons and protons/antiprotons. A summary and an outlook are given in Sect. 4.

## Experimental and theoretical input

In this section, we present the SIA and *pp* data sets used in this work and discuss the theoretical calculation of the corresponding observables.

### The data set

In this analysis we include all available SIA measurements from LEP (ALEPH [40], DELPHI [41, 42] and OPAL [43, 44]), PETRA (TASSO [45]), PEP (TPC [46]) and SLC (SLD [47]). These measurements consist of cross sections differential in the scaling variable $z=2(p^h\cdot q)/Q^2$, where $p^h$ is the four-momentum of the final-state hadron, *q* is the four-momentum of the exchanged virtual gauge boson and $Q\equiv \sqrt{q^2}$. They are normalised to the total cross section for inclusive electron–positron annihilation into hadrons, $\sigma _\mathrm{tot}$. Besides measurements based on inclusive samples, which contain all quark flavours, we also include measurements based on flavour-enriched (or tagged) *uds*-, *c*- and *b*-quark samples from DELPHI [41, 42], OPAL [43] and SLD [47]. This data set is then equivalent to that of the identified charged pions, kaons and protons/antiprotons set used in NNFF1.0. We refer the reader to Ref. [18] for a detailed discussion.

In contrast with identified light hadrons, separate measurements of the longitudinal contribution to the differential cross sections are available for unidentified charged hadrons. We include both inclusive measurements, provided by DELPHI [42] and OPAL [44], and *uds*- and *b*-tagged measurements, provided by DELPHI [42].

The features of the SIA measurements included in this analysis, such as the centre-of-mass energy $\sqrt{s}$, the number of data points for each experiment and their references, are summarised in Table 1 of Ref. [33]. Our SIA data set mostly overlaps that of previous analyses [28–31, 33, 34].

Concerning *pp* data, we include all available measurements from the Tevatron (CDF [9, 10]) and the LHC (ALICE [15] and CMS [13, 14]). They consist of cross sections differential in the momentum of the final-state hadron, $p^h$, presented as a function of its transverse component $p_T^h$ at different centre-of-mass energies $\sqrt{s}$. Specifically, we include CDF data at 1.80 TeV [9] and 1.96 TeV [10], CMS data at 0.9 TeV [13], 2.76 TeV [14] and 7 TeV [13], and ALICE data at 0.9 TeV, 2.76 TeV, and 7 TeV [15]. The covered rapidity range is $|\eta |<1$ for CDF and CMS and $|\eta |<0.8$ for ALICE. The CMS and ALICE data is used here for the first time to constrain FFs.

**Table 1:** The data set included in the NNFF1.1h analysis. For each hadron collider experiment, we indicate the publication reference, the centre-of-mass energy $\sqrt{s}$, the number of data points included after (before) kinematic cuts $N_\mathrm{dat}$, the $\chi ^2$ per number of data points before (after) reweighting, $\chi ^2_\mathrm{in}/N_\mathrm{dat}$ ($\chi ^2_\mathrm{rw}/N_\mathrm{dat}$), the number of effective replicas after reweighting, $N_\mathrm{eff}$, and the modal value of the $\mathcal {P}(\alpha )$ distribution in the range $\alpha \in [0.5,4]$, $\mathrm{argmax}\,\mathcal {P}(\alpha )$. For SIA experiments, see Table 1 in [33]

Process	Experiment	Refs.	$\sqrt{s}$ (TeV)	$N_\mathrm{dat}$	$\chi ^2_\mathrm{in}/N_\mathrm{dat}$	$\chi ^2_\mathrm{rw}/N_\mathrm{dat}$	$N_\mathrm{eff}$	$\mathrm{argmax}\,\mathcal {P}(\alpha )$
SIA	Various, see Table 1 in [33]			471 (527)	0.83	0.83	–	–
*pp*	CDF	[9]	1.80	2 (49)	3.32	0.20	1420	0.49
		[10]	1.96	50 (230)	2.93	1.23	735	1.16
	CMS	[13]	0.90	7 (20)	4.20	0.70	1206	0.96
		[14]	2.76	9 (22)	10.6	1.24	579	0.94
		[13]	7.00	14 (27)	12.4	1.64	396	0.81
	ALICE	[15]	0.90	11 (54)	4.94	1.88	1012	0.93
		[15]	2.76	27 (60)	13.3	0.82	574	0.69
		[15]	7.00	22 (65)	6.03	0.53	779	0.81
				603 (1054)	6.54	1.11	407	1.10

We do not consider older measurements performed by the UA1 [48–50] and UA2 [51] experiments at the $\hbox {Sp} \overline{\mathrm{p}}\hbox {S}$ nor those by the PHENIX experiment [52] at RHIC. These measurements mostly cover the low-$p_T^h$ region, where large missing higher-order corrections affect the theoretical predictions. They would therefore be almost completely excluded by our kinematic cuts (see Sect. 2.2). These measurements were also found to be poorly described when included in a global fit of FFs [31].

The features of our *pp* data set are summarised in Table 1, where we specify the name of each experiment, the publication reference, the centre-of-mass energy $\sqrt{s}$ and the number of data points, $N_\mathrm{dat}$.

### Theoretical calculations

The normalised SIA total (longitudinal) cross section can be expressed in a factorised form as

1 $$\begin{aligned} \frac{1}{\sigma _\mathrm{tot}}\frac{d\sigma ^{h^\pm }_{2(L)}}{dz}(z,Q) = \frac{4\pi \alpha ^2}{\sigma _\mathrm{tot} Q^2}\sum _{l} C_{2(L)}^l(z,Q)\otimes D_l^{h^\pm }(z,Q), \end{aligned}$$

where $h^\pm$ denotes the sum of unidentified charged hadrons, $h^\pm =h^++h^-$, $\alpha$ is the quantum electrodynamics (QED) coupling constant and $\otimes$ represents the convolution product between the perturbative total (longitudinal) coefficient functions $C_{2(L)}^l$ and the non-perturbative FFs $D_l^{h^\pm }$ associated to the parton *l*. The sum over *l* in Eq. (1) runs over all active partons at the scale *Q*.

As discussed in Sect. 3.1 of Ref. [18], the observable defined in Eq. (1) is sensitive only to a limited number of quark FF combinations and to the gluon FF. In the case of the quark FFs, SIA measurements provide limited sensitivity to the separation between the different light-quark FFs, while a direct handle on the separation between light- and heavy-quark FFs is provided by the flavour-tagged data. The gluon FF is poorly constrained by the total SIA cross sections $d\sigma ^{h^\pm }_{2}/dz$. The reason being that the total coefficient function of the gluon, $C_{2}^g$, receives its leading-order (LO) contribution at $\mathcal {O}(\alpha _s)$, while that of the quark, $C_{2}^q$, at $\mathcal {O}(1)$ [53–56]. Conversely, the longitudinal cross section $d\sigma ^{h^\pm }_{L}/dz$ has a comparable sensitivity to gluon and quark FFs because both coefficient functions, $C_{L}^g$ and $C_{L}^q$, start at $\mathcal {O}(\alpha _s)$. Noticeably, measurements of the longitudinal SIA cross section are available only for the production of unidentified hadrons.

The numerical computation of the cross sections in Eq. (1) and of the evolution of FFs is performed at NLO using APFEL [57, 58] as in the NNFF1.0 analysis. In contrast with NNFF1.0, we cannot analyse SIA data at next-to-next-to-leading order (NNLO) as perturbative corrections to the coefficient functions of the longitudinal cross section in Eq. (1) are only known up to $\mathcal {O}(\alpha _s^2)$, i.e. NLO.

To avoid regions where small- and large-*z* resummation effects are sizeable, we impose kinematic cuts on the SIA data. We adopt the same cuts used in the NNFF1.0 analysis, where data points below $z_\mathrm{min}$, with $z_\mathrm{min}=0.02$ for experiments at $\sqrt{s}=M_Z$ and $z_\mathrm{min}=0.075$ for the rest, and above $z_\mathrm{max}=0.9$ are excluded from the fit.

Turning to the differential distribution of the final-state hadron in *pp* collisions, it can be expressed in a factorised form as

2 $$\begin{aligned} E_h\frac{d^3\sigma ^{h^\pm }}{d^3 p^h} = \sum _{i,j,l} K_{ij}^l\otimes f_i(x_1,\mu ) \otimes f_j(x_2,\mu ) \otimes D_l^{h^\pm }(z,\mu ), \end{aligned}$$

where $E_h$ and $p^h$ are the energy and the three-momentum of the produced hadron, $f_i(x_1,\mu )$ and $f_j(x_1,\mu )$ are the PDFs of the colliding hadrons, $D_l^{h^\pm }(z,\mu )$ is the FF of the outgoing hadron, $K_{ij}^l$ are the perturbative hard cross sections and the summation runs over all active partons *i*, *j*, *k* at the scale $\mu$. In principle, the factorisation scale $\mu$ could be chosen independently for PDFs and FFs, and independently from the renormalisation scale used in $\alpha _s$. In practice, our nominal choice is to set all scales equal to the transverse momentum of the produced hadron, *i.e.* $\mu =p_T^h$.

If heavy-quark masses are neglected, as done here, the hard cross sections $K_{ij}^l$ in Eq. (2) are blind to the quark flavour of the FF. This implies that the index *l* distinguishes only whether the outgoing parton is a gluon or a quark, regardless of its flavour. This structure can be made explicit by re-rewriting Eq. (2) as

3 $$\begin{aligned} E_h\frac{d^3\sigma ^{h^\pm }}{d^3 p^h} = \sum _{i,j} f_i \otimes f_j \otimes [K_{ij}^g\otimes D_g^{h^\pm } + K_{ij}^q\otimes D_\Sigma ^{h^\pm }], \end{aligned}$$

where we drop all function dependencies to simplify the notation and define the singlet FF as $D_\Sigma ^{h^\pm }=\sum _q D_q^{h^\pm }+D_{\bar{q}}^{h^\pm }$. The flavour structure of the observable in Eq. (3) is therefore such that *pp* cross-section data is sensitive only to two independent FF combinations, namely $D_g^{h^\pm }$ and $D_\Sigma ^{h^\pm }$. This is a subset of the combinations involved in the computation of the SIA cross sections, see e.g. Eq. (3.1) in Ref. [18]. This property ensures that a prior set of FFs determined from a fit to SIA data only can be sensibly reweighted with *pp* cross section data, as this is not sensitive to any new FF combinations.

The relative contribution of quark and gluon FFs to Eq. (2) depends on the kinematics. It was estimated [32] that at $\sqrt{s}=0.9\ \hbox {TeV}$ ($\sqrt{s}=7\ \hbox {TeV}$) the contribution due to the gluon FF dominates over the quark one in the region $p_T^h\lesssim 20\ \hbox {GeV}$ ($p_T^h\lesssim 100\ \hbox {GeV}$). Therefore, the gluon contribution remains sizeable in most of the kinematic region covered by the *pp* measurements considered in this analysis. For this reason we expect that including *pp* data in a fit will have a significant impact on the gluon FF.

Perturbative corrections to the hard cross sections $K_{ij}^l$ in Eq. (2) are currently known up to $\mathcal {O}(\alpha _s^3)$ [59–62], *i.e.* NLO. Theoretical predictions are computed at this order, consistently with those for SIA data. The numerical computation of Eq. (2) is performed with the code presented in Refs. [61, 62]. Results have been benchmarked against the alternative INCNLO code [59, 63] to a relative precision well below the experimental uncertainties. Parton distributions are taken as an external input from the NLO NNPDF3.1 determination [64]. We do not include PDF uncertainties as it has been previously shown [32] that they are negligible in comparison to FF uncertainties.

At relatively small values of $p_T^h$ ($p_T^h\lesssim 5-10\ \hbox {GeV}$), NLO theoretical predictions for the cross section in Eq. (2) are affected by large uncertainties due to missing higher-order corrections [32]. A kinematic cut $p_{T,\mathrm{cut}}^h$ is therefore imposed to remove all the data with $p_T^h<p_{T,\mathrm{cut}}^h$. In this analysis, we choose $p_{T,\mathrm{cut}}^h=7\ \hbox {GeV}$ as a nominal cut. This value is determined by studying the stability of the FFs and the quality of the fit upon variations of the value of $p_{T,\mathrm{cut}}^h$ in the range $5 \text { GeV} \le p_{T,\mathrm{cut}}^h \le 10\ \hbox {GeV}$ and by varying the scale $\mu$ by a factor of two up and down with respect to our central choice, $\mu =p_T^h$, see Sect. 3.2.

## Results

In this section we present the results of our analysis. First, we describe how the experimental and theoretical inputs described in Sect. 2 are combined to construct our set of FFs, dubbed NNFF1.1h. We present the fit quality and compare the input data set to the corresponding theoretical predictions, focusing on the impact of hadron-collider measurements. Then, we motivate our choice of the value of $p_{T,\mathrm{cut}}^h$ by investigating the stability of the fit upon variations of $p_{T,\mathrm{cut}}^h$ and of the scale $\mu$ used to compute the hadron-collider cross sections. Finally, we study the consistency of the NNFF1.1h set with the NNFF1.0 sets for identified pion, kaon and proton/antiproton FFs.

### The NNFF1.1h set

In this analysis, we determine the FFs of unidentified charged hadrons in two steps. In the first step, we construct a set of $N_\mathrm{rep}=2000$ equally probable Monte Carlo FF replicas from a fit to the SIA data presented in Sect. 2.1. In the second step, we use this set as a prior to include the *pp* data presented in Sect. 2.1 by means of Bayesian reweighting [38, 39]. The reweighted set is then unweighted to produce an ensemble of $N_\mathrm{rep}=100$ equally probable Monte Carlo FF replicas. This set forms our final deliverable result, NNFF1.1h.

**Fig. 1:**
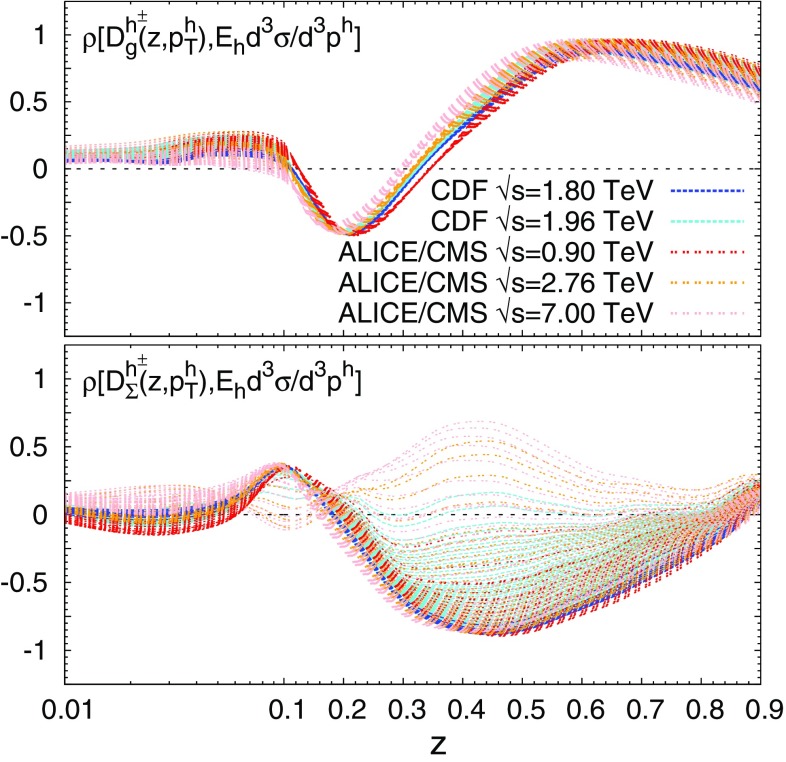
The correlation coefficient $\rho$ between the gluon (top) and the singlet (bottom) FFs from NNFF1.0h and the *pp* data listed in Table 1. Each data point corresponds to a separate curve; FFs are evaluated at a scale $\mu$ equal to the $p_T^h$ of that point

The initial fit to SIA data closely follows the NNFF1.0 analysis, the methodological details of which are extensively discussed in Sects. 4.1 and 4.3 of Ref. [18]. The results of this fit, which we here call NNFF1.0h, were presented in Ref. [33]. The NNFF1.0h set provides a good description of its dataset, with a total $\chi ^2$ per data point of $\chi _\mathrm{in}^2/N_\mathrm{dat}=0.83$ for $N_\mathrm{dat}=471$ data points (note that henceforth we will use the subscript “$_\mathrm{in}$” whenever a $\chi ^2$ is computed with NNFF1.0h). The values for the individual SIA experiments included in NNFF1.0h can be found in Table 1 of Ref. [33]. A data/theory comparison is reported in Fig. 1 of the same reference.

The NNFF1.0h set is then used to produce the theoretical predictions for the *pp* data discussed in Sect. 2.1 according to the details presented in Sect. 2.2. The resulting values of $\chi ^2_\mathrm{in}/N_\mathrm{dat}$ for each experiment are reported in Table 1. The corresponding data/theory comparison is displayed in Figs. 3, 4 and 5. The $\chi ^2$ values in Table 1 are computed using the full covariance matrix, constructed from all the uncorrelated and correlated experimental uncertainties. For illustration the uncertainty bars shown in Figs. 3, 4 and 5 are the sum in quadrature of only the uncorrelated uncertainties. The effect of the correlated systematic uncertainties is taken into account (assuming a Gaussian distribution) by shifting the theoretical predictions [65]. While this shift facilitates a qualitative assessment of the data/theory agreement, the quality of the fit can only be precisely judged from the $\chi ^2$ values reported in Table 1.

As is apparent from Table 1, the agreement between the *pp* data and the theoretical predictions obtained with the NNFF1.0h set is not particularly good. The values of $\chi ^2_\mathrm{in}/N_\mathrm{dat}$ range from around 3 for the CDF data up to 13.3 for the ALICE data at $\sqrt{s}=2.76\ \hbox {TeV}$. However, from Figs. 3, 4 and 5 we see that theoretical predictions are affected by uncertainties due to FFs (not included in the $\chi ^2$ computation) much larger than the uncertainty of the data. If FF uncertainties are taken into account, the calculations based on NNFF1.0h agree with the data at the one-$\sigma$ level. This suggests that the *pp* data is consistent with the SIA data used to determine NNFF1.0h and that, at the same time, it should be able to significantly constrain unidentified charged-hadron FFs.

The region of the momentum fraction *z* for which the hadron-collider data has potentially the largest impact on the FFs can be quantified by computing the correlation coefficient $\rho$ (see Eq. (1) in Ref. [66] for its definition) between the FFs in the NNFF1.0h set and the theoretical predictions corresponding to the *pp* data sets discussed in Sect. 2.1. Large values of $|\rho |$ indicate regions in *z* where the sensitivity of FFs to the data is most significant. The correlation coefficient $\rho$ is displayed in Fig. 1 for the gluon and singlet FFs. Each curve corresponds to a different data point; FFs are evaluated at the scale $\mu$ equal to the $p_T^h$ of that point. We observe that the correlation is maximal for $z\gtrsim 0.4$ in the case of the gluon FF for almost all data points and for $0.2\lesssim z\lesssim 0.7$ in the case of the singlet FF, although for a more limited number of data points. The sensitivity is negligible for $z\lesssim 0.1$ in both cases.

The *pp* data listed in Table 1 is used to constrain the NNFF1.0h set by means of Bayesian reweighting [38, 39]. This method consists in updating the representation of the probability density in the space of FFs by means of Bayes’ theorem. Specifically, each replica of the NNFF1.0h set is assigned a weight that quantifies its agreement with the new data. These weights are computed by evaluating the $\chi ^2$ of the new data using the predictions obtained with that given replica. After reweighting, replicas with smaller weights become less relevant in ensemble averages, therefore the number of effective replicas in the Monte Carlo ensemble is reduced. The consistency of the data used for reweighting with the prior can be assessed by examining the $\mathcal {P}(\alpha )$ profile of the new data, where $\alpha$ is the factor by which the uncertainty of the new data must be rescaled in order for both the prior and the reweighted sets to be consistent with each other. If the modal value of $\alpha$ is close to unity, the new data is consistent with the original one within the quoted experimental uncertainties.

We construct the NNFF1.1h set by reweighting the NNFF1.0h set simultaneously with all the *pp* data listed in Table 1. The values of the $\chi ^2$ per data point after reweighting, $\chi ^2_\mathrm{rw}/N_\mathrm{dat}$, the number of effective replicas, $N_\mathrm{eff}$, and the modal value of the $\mathcal {P}(\alpha )$ distribution in the region $\alpha \in [0.5,4]$, $\mathrm{argmax}\,\mathcal {P}(\alpha )$, are also collected in Table 1.

The value of the $\chi ^2$ per data point for the *pp* data decreases significantly after reweighting for all experiments down to values of order one. The improvement is particularly marked for the CMS and ALICE data, where experimental uncertainties are smaller than those for CDF. The description of the SIA data is not affected by the inclusion of the *pp* data in the fit, since the corresponding $\chi ^2$ remains unchanged. We explicitly checked that this is true also for the individual SIA experiments. This confirms that there is no tension between the new *pp* measurements and the SIA data used in NNFF1.0h.

The number of effective replicas after reweighting depends significantly on the specific data set: in general, the more precise the data set, the smaller the number of effective replicas. The total size of the reweighted FF set, made of $N_\mathrm{eff}=407$ effective replicas, is around 20% of the size of the prior set, composed of $N_\mathrm{rep}=2000$ replicas. This number is sufficiently large to ensure an adequate statistical accuracy of the unweighted FF set, since it is significantly larger than $N_\mathrm{rep}=100$, the customary number of replicas of a typical NNPDF set. The reweighted set is then finally unweighted into $N_\mathrm{rep}=100$ equally probable replicas to construct the NNFF1.1h set.

The modal value of the $\mathcal {P}(\alpha )$ distribution in the region $\alpha \in [0.5,4]$, $\mathrm{argmax}\,\mathcal {P}(\alpha )$, is of order one for all *pp* data sets. This is a further confirmation of the consistency within the quoted experimental uncertainties of the *pp* and SIA data sets included in this analysis.

**Fig. 2:**
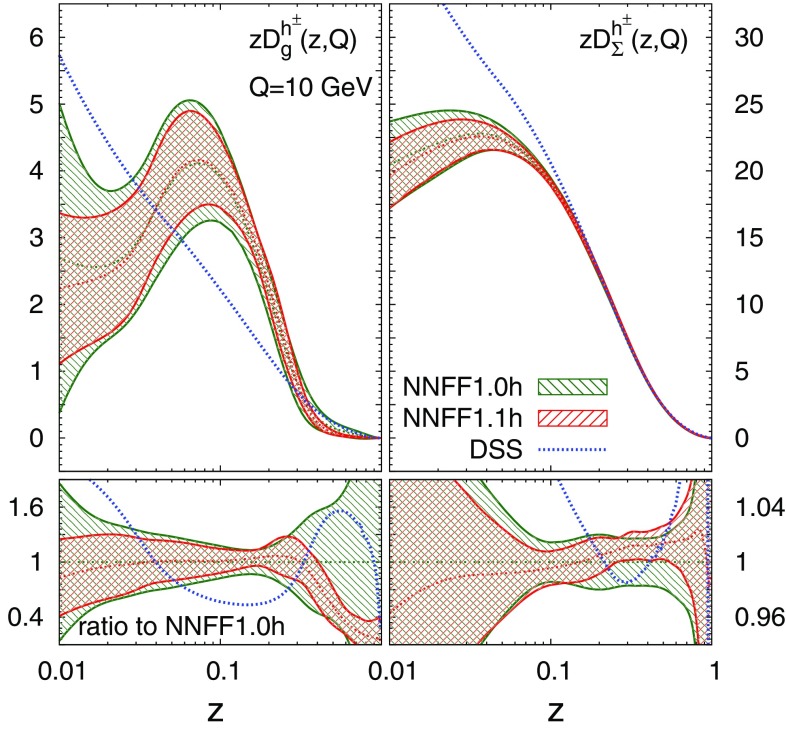
The gluon (left) and singlet (right) FFs for the unidentified charged hadrons from NNFF1.0h, NNFF1.1h, and DSS at $Q=10\ \hbox {GeV}$; the bands indicate the one-$\sigma$ uncertainties. The ratio to NNFF1.0h is displayed in the bottom panels

**Fig. 3:**
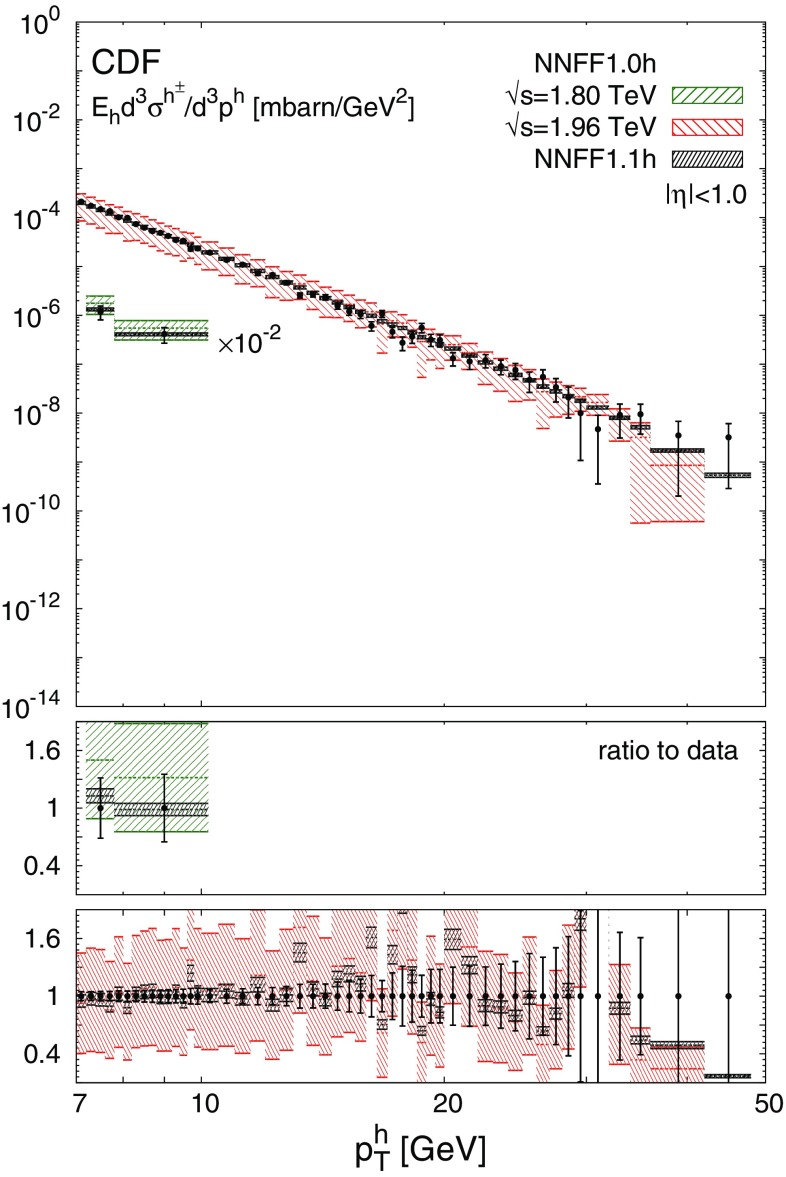
The differential cross section, Eq. (2), for the inclusive charged hadron spectra measured by CDF in proton-antiproton collisions at different centre-of-mass energies over the rapidity range $|\eta |<1$. The data is compared to the NLO predictions obtained with NNFF1.0h and NNFF1.1h. The corresponding theory/data ratio is shown in the lower panels. The bands include the one-$\sigma$ FF uncertainty only. We show the sum in quadrature of the uncorrelated uncertainties on the data points, while correlated systematic errors are taken into account via shifts of the theoretical predictions (see text)

**Fig. 4:**
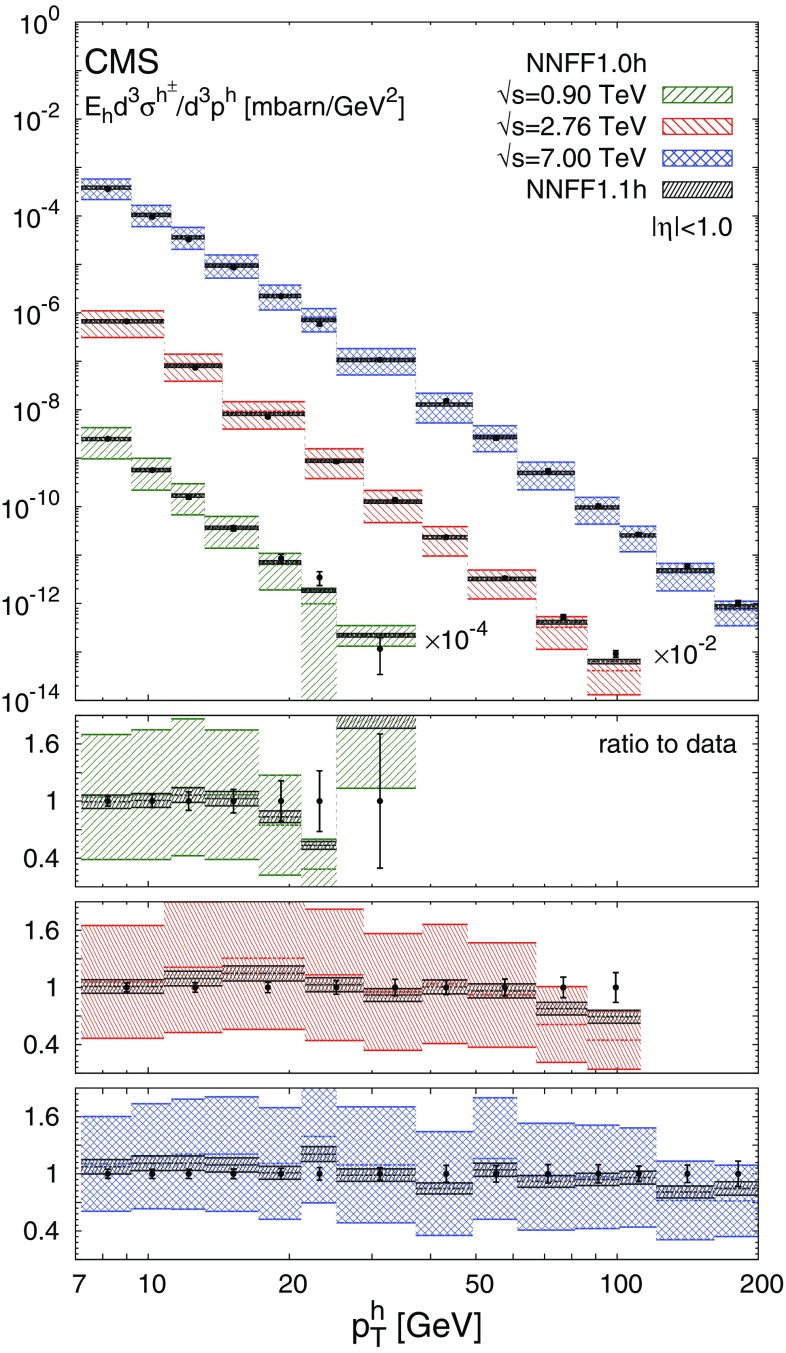
Same as Fig. 3 for the (proton-proton) CMS data sets

**Fig. 5:**
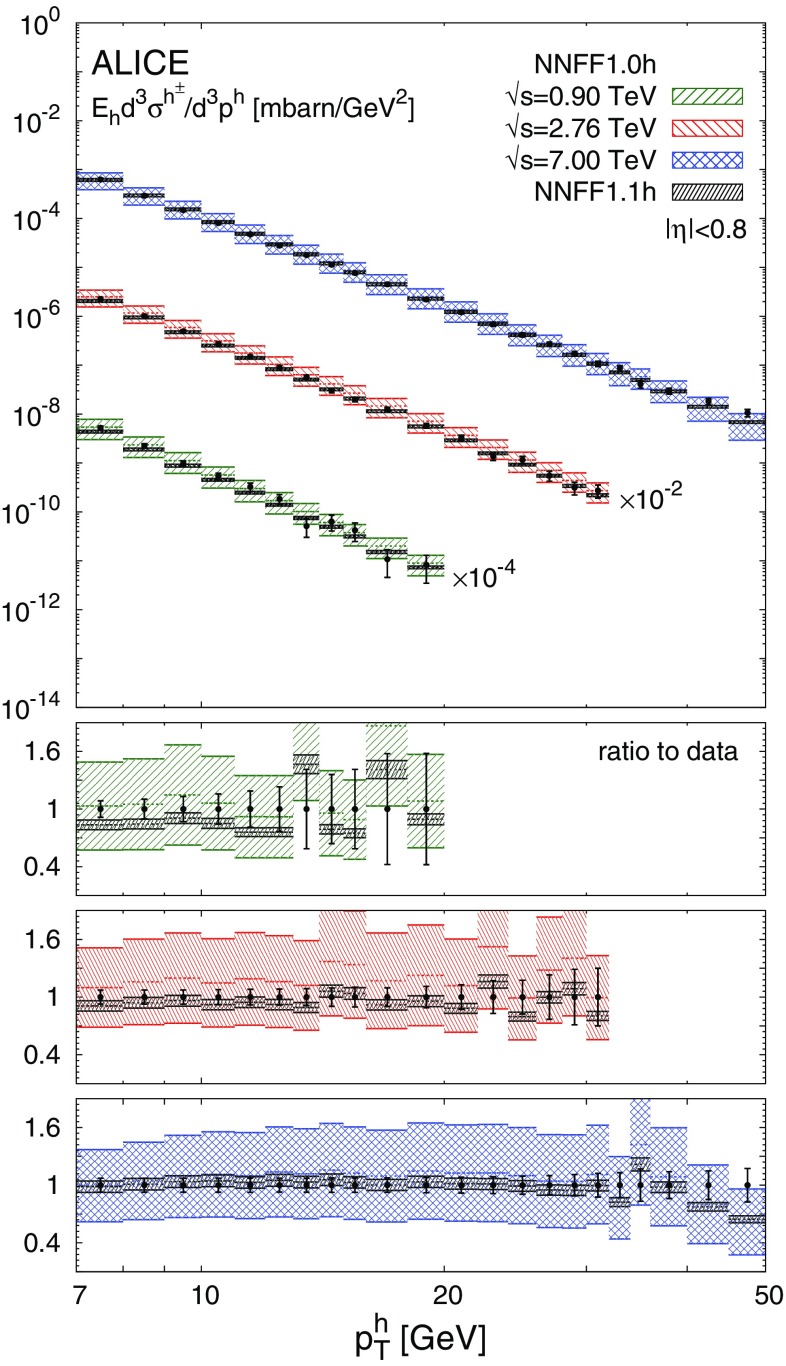
Same as Fig. 4 for the ALICE data sets

The gluon and singlet FFs from NNFF1.1h at $Q=10\ \hbox {GeV}$ are shown in Fig. 2. They are compared to the corresponding FFs from the NNFF1.0h and the DSS [31] sets. The ratio to NNFF1.0h is displayed in the bottom panel. The theoretical predictions for the *pp* data obtained with NNFF1.1h are shown in Figs. 3, 4 and 5 on top of their counterparts obtained from NNFF1.0h.

As is apparent from Table 1 and Figs. 2, 3, 4 and 5, the impact of the *pp* data on the FFs is twofold. First, it induces a modification of the shape of the FFs. The central value of the gluon FF moves towards slightly harder values in the region $0.1\lesssim z \lesssim 0.3$ and towards significantly softer values in the region $0.3\lesssim z \lesssim 0.9$. The central value of the singlet FF remains stable except in the region $0.1\lesssim z \lesssim 0.4$, where it becomes slightly smaller. Second, the *pp* data leads to a significant reduction of the FF uncertainties. For the gluon FF the relative uncertainty drops from 20–60 to 10–15% in the region $z\gtrsim 0.1$, i.e. a reduction of up to a factor four. For the singlet FF which is already well constrained by SIA data, the reduction is more moderate but still significant, with the uncertainty decreasing in the region $0.1\lesssim z \lesssim 0.4$ from around 2% to $\simeq 1\%$. Both the shape and the uncertainties of the gluon and singlet FFs are almost unchanged for $z\lesssim 0.07$, as expected from the correlations between *pp* data and FFs shown in Fig. 1. The NNFF1.1h uncertainty bands are encapsulated by those of NNFF1.0h. This further confirms the good consistency between SIA and *pp* measurements included in our analysis.

Finally, we note that the central value of the gluon and singlet FFs of the NNFF1.1h set is quite different from that of the DSS set. Specifically, the gluon and singlet FFs are harder in NNFF1.1h than in DSS for $0.03\lesssim z\lesssim 0.3$ but softer elsewhere. No estimate of the FF uncertainties was determined in the DSS fit, hence it is not possible to quantitatively assess its statistical compatibility with our results. The fact that hadron-collider cross sections prefer a softer gluon FF at large-*z* was already suggested in Ref. [32] as a possible explanation of the poor agreement between *pp* data and theory predictions when the latter is computed with DSS.

### Dependence on the value of $p_{T,\mathrm{cut}}^h$

Having presented the impact of the *pp* data on FFs, we now provide a rationale for our choice of the baseline cut on the hadron transverse momentum, $p_{T,\mathrm{cut}}^h = 7\ \hbox {GeV}$. This is motivated by examining the dependence of our study upon this cut in the range $5\ \hbox {GeV}$ $\le p_{T,\mathrm{cut}}^h \le 10 \ \hbox {GeV}$ with steps of $1\ \hbox {GeV}$. This range of $p_{T,\mathrm{cut}}^h$ values being chosen in accordance with the study of Ref. [32], where it was shown that in this range theoretical uncertainties due to missing higher-order corrections become sizeable. In Table 2, we collect the number of data points after the cut and the corresponding $\chi ^2_\mathrm{rw}/N_\mathrm{dat}$ values after the *pp* data set is used to reweight the NNFF1.0h set.

The fits with the most restrictive cuts, $p_{T,\mathrm{cut}}^h=9\ \hbox {GeV}$ and $p_{T,\mathrm{cut}}^h=10\ \hbox {GeV}$, naturally have a number of data points rather smaller than those with the less conservative cut, $p_{T,\mathrm{cut}}^h=5\ \hbox {GeV}$. Most notably, no points of the $\sqrt{s}= 1.80\ \hbox {TeV}$ CDF data set pass these cuts.

As one may expect the overall fit quality deteriorates, albeit modestly, if a larger number of low-$p_T^h$ points is included in the fit. In particular, the total $\chi ^2_\mathrm{rw}/N_\mathrm{dat}$ of the *pp* data sets increases from 1.08 for $p_{T,\mathrm{cut}}^h=10\ \hbox {GeV}$ to 1.27 for $p_{T,\mathrm{cut}}^h=5\ \hbox {GeV}$. The description of almost all data sets is worse or significantly worse in the fit with $p_{T,\mathrm{cut}}^h=5\ \hbox {GeV}$ than in that with $p_{T,\mathrm{cut}}^h=10\ \hbox {GeV}$. For the CMS 7 TeV and ALICE 0.9 TeV data sets, the $\chi ^2_\mathrm{rw}/N_\mathrm{dat}$ increases from 1.40 and 1.52 to 2.01 and 2.56, respectively, when one lowers the cut from $10\ \hbox {GeV}$ to $5\ \hbox {GeV}$. The description of the ALICE 2.76 TeV and 7 TeV data is instead moderately better with $p_{T,\mathrm{cut}}^h=5\ \hbox {GeV}$ than the one with $p_{T,\mathrm{cut}}^h= 10\ \hbox {GeV}$.

**Table 2:**
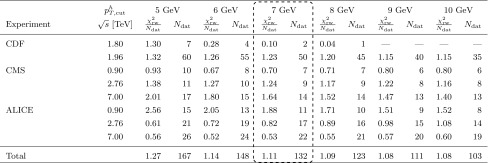
The values of the $\chi ^2$ per data point, $\chi ^2_\mathrm{rw}/N_\mathrm{dat}$, and the number of data points after cuts, $N_\mathrm{dat}$, for the *pp* experiments included in the fit (and their total) for a range of values of the kinematic cut $p_{T,\mathrm{cut}}^h$. Our baseline is $p_{T,\mathrm{cut}}^h=7\ \hbox {GeV}$

The overall fit quality turns out to be very similar for values of $p_{T,\mathrm{cut}}^h$ larger or equal to 6 GeV. Conversely, it markedly worsens when we lower the value of $p_{T,\mathrm{cut}}^h$ from $6\ \hbox {GeV}$ to $5\ \hbox {GeV}$. In this case, the $\chi ^2_\mathrm{rw}/N_\mathrm{dat}$ increases from 1.14 to 1.27, mostly because of the poor description of the 1.8 TeV CDF data set, whose $\chi ^2_\mathrm{rw}/N_\mathrm{dat}$ raises from 0.28 to 1.30. A deterioration is also observed in the $\chi ^2_\mathrm{rw}/N_\mathrm{dat}$ of almost all the other data sets; in particular, it increases from 0.67 to 0.93 and from 2.05 to 2.56 for the 0.9 TeV CMS and ALICE data sets respectively.

This study of the fit quality suggests that reliable results require a value of $p_{T,\mathrm{cut}}^h \ge 6\ \hbox {GeV}$. To find the optimal value of $p_{T,\mathrm{cut}}^h$ in the restricted range 6 GeV $\lesssim p_{T,\mathrm{cut}}^h\lesssim \ 10\ \hbox {GeV}$, we investigate the perturbative stability of the FFs by repeating the reweighting procedure with the scale $\mu$ in Eq. (2) set to $2 p_T^h$ and $p_T^h/2$. We then study the behaviour of the resulting FFs for different values of $p_{T,\mathrm{cut}}^h$. We find that FFs are reasonably stable with respect to variations of the scale $\mu$ if $p_{T,\mathrm{cut}}^h$ is equal to 7 GeV or larger, whereas the same variations lead to larger distortions in shape for $p_{T,\mathrm{cut}}^h=6\ \hbox {GeV}$.

**Fig. 6:**
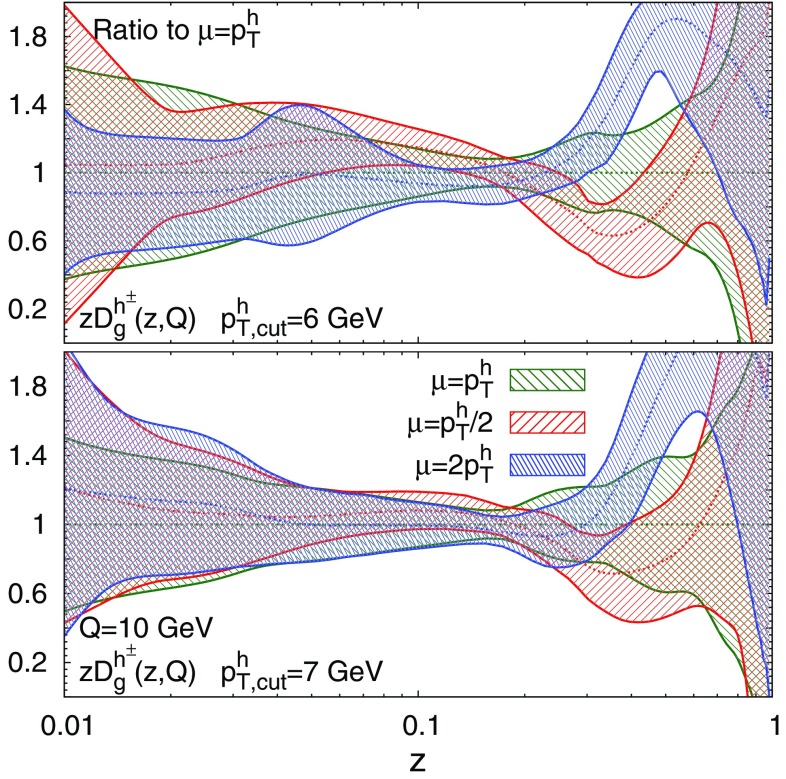
Comparison of the gluon FF at $Q= 10\ \hbox {GeV}$ for the fits performed setting the scale $\mu$ in Eq. (2) to $p_T^h$, $2 p_T^h$ or $p_T^h/2$ for $p_{T,\mathrm{cut}}^h=6\ \hbox {GeV}$ (upper) and the baseline $p_{T,\mathrm{cut}}^h=7\ \hbox {GeV}$ (lower plot), normalised to the $\mu =p_T^h$ result

To illustrate this, in Fig. 6 we show a comparison of the gluon FF for $p_{T,\mathrm{cut}}^h=6\ \hbox {GeV}$ and $p_{T,\mathrm{cut}}^h=7\ \hbox {GeV}$ at $Q=10\ \hbox {GeV}$ for the fits performed setting the scale $\mu$ to $p_T^h$, $2 p_T^h$, and $p_T^h/2$, normalised to the nominal $\mu =p_T^h$ result. We observe that in the $p_{T,\mathrm{cut}}^h=6\ \hbox {GeV}$ case, for values of *z* between 0.1 and 0.5, the two uncertainty bands of the FFs with $\mu =2p_T^h$ and $\mu =p_T^h/2$ do not overlap, and that their central value is not contained in the band of the FFs obtained using the central scale $\mu =p_T^h$. This discrepancy is partially reduced with $p_{T,\mathrm{cut}}^h=7\ \hbox {GeV}$ and we checked that the fit with $p_{T,\mathrm{cut}}^h=10\ \hbox {GeV}$ has a similar pattern. This behaviour is also exhibited by the singlet FF. We conclude that by choosing $p_{T,\mathrm{cut}}^h=6\ \hbox {GeV}$ one would add to the fit data points that may not be described reliably using NLO QCD theory. Therefore, this motivates our baseline choice $p_{T,\mathrm{cut}}^h = 7\ \hbox {GeV}$.

**Fig. 7:**
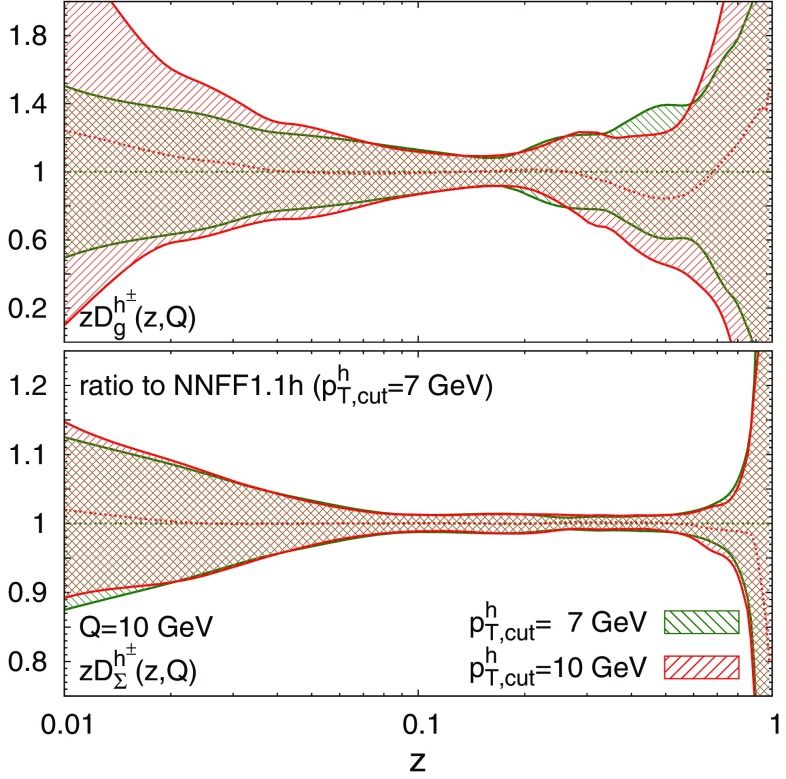
Comparison of the gluon (upper) and singlet (lower plot) FFs at $Q=10\ \hbox {GeV}$ for the NNFF1.1h fits with $p_{T,\mathrm{cut}}^h=7\ \hbox {GeV}$ and $p_{T,\mathrm{cut}}^h=10\ \hbox {GeV}$, normalised to the former

As further evidence in favour of our choice of $p_{T,\mathrm{cut}}^h$, in Fig. 7 we compare the gluon and singlet FFs at $Q=10\ \hbox {GeV}$ from the fit with our default choice $p_{T,\mathrm{cut}}^h = 7\ \hbox {GeV}$ to those obtained with the more restrictive $p_{T,\mathrm{cut}}^h = 10\ \hbox {GeV}$, normalised to the former. In both cases the resulting FFs are similar and the central value of the $p_{T,\mathrm{cut}}^h = 7\ \hbox {GeV}$ fit is always contained within the uncertainty band of the $p_{T,\mathrm{cut}}^h = 10\ \hbox {GeV}$ fit. This comparison shows that the two fits are compatible and demonstrates the reliability of the fit upon our nominal choice of $p_{T,\mathrm{cut}}^h$.

In summary, the study of the fit quality and of the stability of FFs with respect to scale variations suggests that the choice $p_{T,\mathrm{cut}}^h = 7\ \hbox {GeV}$ is reasonably optimal: it allows us to include in the fit a sufficiently large number of data points and at the same time it guarantees that the fit is not significantly affected by missing higher-order corrections.

### Compatibility with NNFF1.0

For each parton *i*, the FFs of unidentified charged hadrons, $D_i^{h^\pm }$, can be regarded as the sum of the FFs of charged pions, $D_i^{\pi ^\pm }$, charged kaons, $D_i^{K^\pm }$, protons and antiprotons, $D_i^{p/\bar{p}}$, and a residual component, $D_i^\mathrm{res^\pm }$, which accounts for heavier charged hadrons, such that

4 $$\begin{aligned} D_i^{h^\pm }=D_i^{\pi ^\pm }+D_i^{K^\pm }+D_i^{p/\bar{p}}+D_i^\mathrm{res^\pm }. \end{aligned}$$

Therefore, cross sections for unidentified charged hadrons can be expressed as the sum of individual cross sections computed with $\pi ^\pm$, $K^\pm$, $p/\bar{p}$ and residual FFs.

**Fig. 8:**
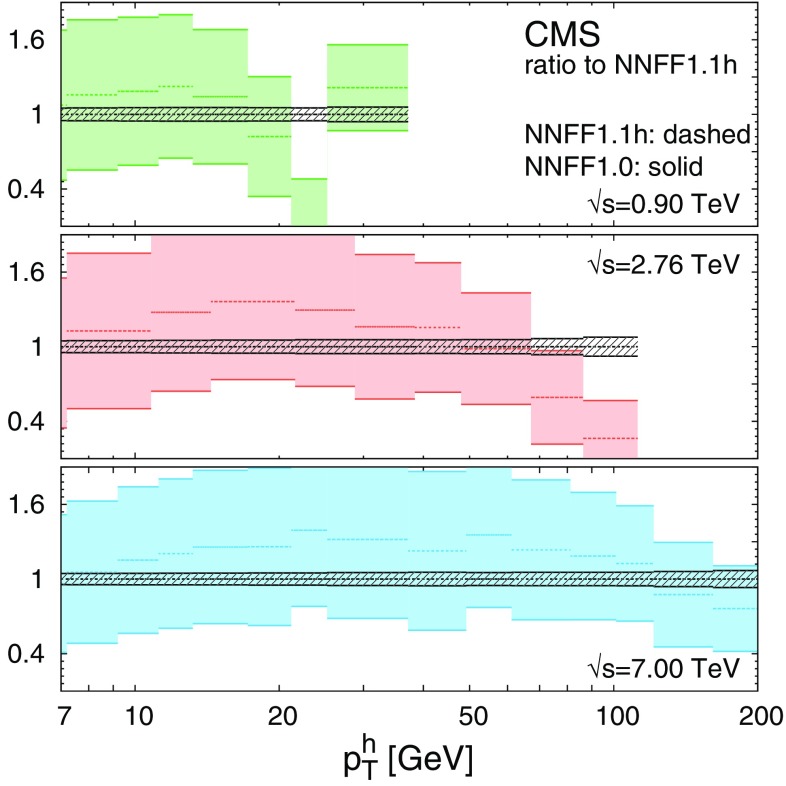
Theoretical predictions for the differential cross sections in *pp* collisions, Eq. (2), computed at NLO in the kinematic bins measured by CMS. We compare the predictions obtained from the unidentified charged hadron in the NNFF1.1h set with those obtained from the sum of charged pions, kaons and protons/antiprotons in the NNFF1.0 set. Predictions are normalised to NNFF1.1h

In this work we do not use Eq. (4) as a theoretical constraint to our FF analysis, as done, for instance, in Ref. [31]. The FFs for unidentified charged hadrons in NNFF1.1h are determined independently from the FFs of identified pions, kaons and protons/antiprotons previously obtained in NNFF1.0. It is therefore interesting to check their consistency. We do so by verifying that the *pp* cross section in Eq. (2) satisfies, within FF uncertainties, the inequality

5 $$\begin{aligned} E_h\frac{d^3\sigma ^{h^\pm }}{d^3p^h} > \sum _{\mathcal {H} =\pi ^\pm ,K^\pm ,p/\bar{p}}E_h\frac{d^3\sigma ^{\mathcal {H}}}{d^3p^h}, \end{aligned}$$

which follows from the positivity of cross sections. In Fig. 8, we compare the l.h.s. and the r.h.s. of Eq. (5), computed at NLO with the FFs from NNFF1.1h and NNFF1.0, respectively, and, as a representative example, for the kinematics of the CMS data. The bands in Fig. 8 correspond to one-$\sigma$ FF uncertainties. We assume that FFs for individual hadronic species are uncorrelated, therefore the uncertainties for the r.h.s. of Eq. (5) are determined by adding in quadrature the uncertainties from the pion, kaon and proton/antiproton NNFF1.0 sets.

The comparison in Fig. 8 shows that the inequality in Eq. (5) is always satisfied within the large uncertainties of the NNFF1.0 result. This also suggests that FF uncertainties for individual hadronic species can be significantly reduced if the corresponding *pp* data are used in their determination.

The consistency between NNFF1.1h and NNFF1.0 can be further assessed in a complementary way by computing the momentum carried by all charged hadrons produced in the fragmentation of the parton (or combination of partons) *i* and by comparing it to the same quantity computed using pions, kaons and protons/antiprotons only. The following relation should then hold within uncertainties:

6 $$\begin{aligned} M_i^{h^\pm }(Q)\equiv & {} \displaystyle \int _{z_\mathrm{min}}^1dz\, z D_i^{h^\pm }(z,Q) \gtrsim \nonumber \\ M_i^\mathrm{light}(Q)\equiv & {} \sum _{\mathcal {H}=\pi ^\pm ,K^\pm ,p/\bar{p}}\int _{z_\mathrm{min}}^1dz\, z D_i^{\mathcal {H}}(z,Q). \end{aligned}$$

According to the same argument given around Eq. (5), the momentum carried by heavier charged hadrons has to be positive. However, contrary to Eq. (5), the inequality does not have to be strictly fulfilled as the integration over *z* in Eq. (6) is truncated at $z_\mathrm{min}$ due to the impossibility of determining FFs down to very small values of *z*. Therefore these (truncated) momentum fractions are not guaranteed to be strictly positive.

We compute $M_i^{h^\pm }(Q)$ and $M_i^\mathrm{light}(Q)$ in Eq. (6) using $z_\mathrm{min}=0.01$ and $Q=5\ \hbox {GeV}$ for NNFF1.1h and NNFF1.0 for charged pions, charged kaons, and protons/antiprotons. The uncertainty of $M_i^\mathrm{light}(Q)$ is determined by adding in quadrature the uncertainties obtained from the single NNFF1.0 sets. The resulting momentum fraction of the gluon FF and the $u^+$, $d^++s^+$, $c^+$ and $b^+$ combinations of quark FFs, with $q^+\equiv q+\overline{q}$, are reported in Table 3. For all the parton combinations considered, $M_i^{h^\pm }(Q)$ and $M_i^\mathrm{light}(Q)$ are compatible within the FF errors, hence the inequality in Eq. (6) is not violated. We therefore conclude that the NNFF1.1h and NNFF1.0 sets are consistent.

We note that the uncertainties of the truncated moments computed with NNFF1.1h are about a factor of three smaller than those obtained with NNFF1.0. This reduction highlights once more the significant constraining power of the *pp* data on the FFs. Additionally, the central value of $M_i^{h^\pm }$ is in general only slightly larger than that of $M_i^\mathrm{light}$ (except for $u^+$ and $d^++s^+$). This suggests that the momentum fraction carried by charged hadrons other than pions, kaons and protons/anti-protons is small and within the uncertainties of NNFF1.1h.

**Table 3:** The momentum fraction, Eq. (6), for the gluon, $u^+$, $d^++s^+$, $c^+$ and $b^+$ FF combinations computed at $Q=5\ \hbox {GeV}$ and $z_\mathrm{min}=0.01$ for the unidentified charged hadron FFs from NNFF1.1h and for the sum of charged pion, kaon and proton/antiproton FFs from NNFF1.0

$Q=5\text { GeV}$	NNFF1.1h	NNFF1.0
*i*	$M_i^{h^\pm }(Q)$	$M_i^\mathrm{light}(Q)$
*g*	$0.86\pm 0.06$	$0.80\pm 0.18$
$u^+$	$1.24\pm 0.07$	$1.42\pm 0.12$
$d^++s^+$	$2.05\pm 0.08$	$2.07\pm 0.27$
$c^+$	$1.09\pm 0.03$	$1.01\pm 0.08$
$b^+$	$1.06\pm 0.02$	$0.98\pm 0.08$

## Summary and outlook

In this work we presented NNFF1.1h, a new determination of the FFs of unidentified charged hadrons based on a comprehensive set of SIA and *pp* measurements. Our study demonstrates that all the data can be simultaneously very well described and that *pp* data significantly constrains the so far poorly known gluon FF. The robustness of NNFF1.1h against potentially large missing higher-order perturbative corrections in the *pp* predictions was ensured by appropriate kinematic cuts. Specifically, the reliability of our results upon our choice of the kinematic cut on the hadron transverse momentum was explicitly verified. We also demonstrated that the NNFF1.1h set is consistent with our previous NNFF1.0 sets for identified charged pions, kaons and protons/antiprotons. Given the high precision of its gluon FF, the NNFF1.1h set could be used to compute theoretical predictions for single-inclusive hadron production in proton-ion and ion-ion collisions, where gluon fragmentation also dominates.

Our work could be extended in at least three directions. First, the charged hadrons SIDIS multiplicities measured by the COMPASS Collaboration [67, 68] could be included in our analysis of unidentified charged-hadron FFs in order to achieve flavour separation. This is possible thanks to the sensitivity of the SIDIS observable to different FF combinations as compared to SIA and *pp*.

Second, this analysis could be repeated for the identified hadronic species determined in NNFF1.0. This would be particularly well motivated in view of the increasing amount of precise data becoming available from LHC experiments [69–71]. These measurements will complement the existing data from RHIC [11, 12, 72–74], part of which, however, comes from longitudinally polarised *pp* collisions. Including data on charged pion, kaon and proton production from the LHC should lead to an improved determination of their gluon FF in the large-*z* region, as is the case for unidentified charged hadrons.

Finally, possible future work is motivated by the realisation that, as shown in this analysis, the LHC data significantly improves the precision with which FFs can be determined. At this point, theoretical uncertainties on hadron-collider cross sections, such as those from missing higher orders, can become comparable in size to the experimental uncertainties. The calculation of NNLO QCD corrections to the *pp* cross sections will therefore be of increasing importance. While such calculations are currently unavailable, they may emerge through the work recently carried out for jet production [75–78]. Meanwhile, our analysis could be extended by taking into account other sources of uncertainty, such as PDF uncertainties, following the procedure outlined in Ref. [79].

The NNFF1.1h set presented in this work is available through the LHAPDF6 interface [80], where the required flavour separation is generated according to the procedure for kaons described in Appendix A of Ref. [18].
